# GC-MS profiling, *in vitro* antioxidant and toxicity evaluation, and *in silico* pharmacokinetic and molecular docking prediction of the phytocompounds of *Rungia repens* (L.) *Nees* targeting SarA of *Staphylococcus aureus*

**DOI:** 10.3389/fchem.2026.1778720

**Published:** 2026-07-22

**Authors:** Vaidagi Balaji, Gayathri Mahalingam

**Affiliations:** School of Bio-Sciences and Technology (SBST), Vellore Institute of Technology (VIT), Vellore, Tamilnadu, India

**Keywords:** adsorption, distribution, metabolism, and excretion, 2,2-diphenyl-1-picrylhydrazyl, GC-MS, L929 cell line, Sar A

## Abstract

The medicinal plant *Rungia repens (L.) Nees* (Acanthaceae) is commonly known as *kodagasalai* in Tamil. It is recognized for its antipyretic, diuretic, anti-inflammatory, and vermifunge properties. The present study is primarily focused on identifying the phytocompounds and evaluating the antioxidant activity and cytotoxicity of *R. repens* leaf extract. *In silico* molecular docking studies were performed for SarA, an anti-virulence target of *Staphylococcus aureus*, and pharmacokinetic prediction was carried out to identify a suitable compound for drug delivery. The total flavonoid (TFC), phenolic content (TPC), and antioxidant activity of the ethanol extract were found to be 65.31 ± 7.2 mg QUE/g, 202.40 ± 2.97 mg GAE/g, and 76.166% ± 0.15% radical-scavenging activity, respectively, with an IC_50_ value of 311.9 ± 1.27 μg/mL. The extract exhibited no toxic effects up to 250 μg/mL against L929 cell line. GC-MS analysis identified 19 phytoconstituents in the *R. repens* ethanol leaf extract. Antibacterial activity against *S. aureus* exhibited an inhibition zone of 14 ± 1.73 mm. Phthalic acid, butyl undecyl ester showed a binding energy of −10.0 kcal/mol against SarA of *S. aureus*. ADME prediction revealed suitable phytocompounds of *R. repens* for drug development. Overall, the results indicate that *R. repens* has abundant compounds with biological activity and offers a source for drug development and delivery strategies.

## Graphical Abstract

**Figure:**
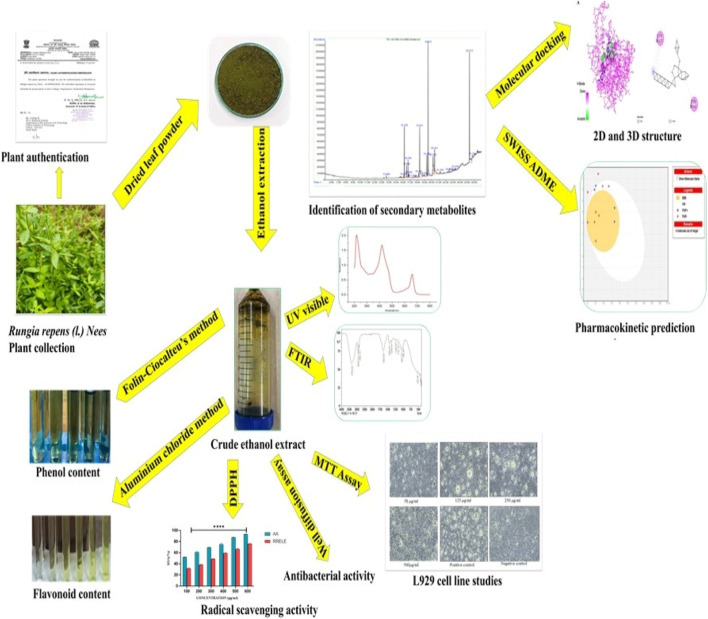

## 1 Introduction

Plants are a wellspring of therapeutic compounds as they have a diverse range of biological activities (Agidew, 2022; Mansoori et al., 2020). Since ancient times, plant-based medicines have had a major impact on human health. Plants and their secondary metabolites, including terpenoids, flavonoids, and phenol, are known for their anticancer, anti-inflammatory, antioxidant, antimalarial, cholesterol synthesis curbing, anti-viral, and antibacterial activities and act as anesthetic drugs (Khalid, 2010; Nandha Kumar et al., 2019; Swain et al., 2008). Plants have gained prominence in recent decades for their low toxicity, safety, and ecological compatibility (Mansoori et al., 2020). Though their non-toxic effects have been evident, their phytochemical profile remains unexplored (Siddique et al., 2021). The chemical ingredients and prospective therapeutic benefits of some known medicinal plant species worldwide have been evaluated effectively (Ouandaogo et al., 2023). More recently, research has concentrated on identifying plant compounds that aid the efficacy of contemporary antibiotics, chiefly as proxies that can counter drug-resistant bacterial strains, rather than only revising interactions within plant extracts. Several medicinal plants have an extensive diversity of pharmacological properties, according to a recent review (Chassagne et al., 2021).

*Rungia repens (L.) Nees*, a member of the Acanthaceae family, is well known in Tamil as *kodagasalai*. It produces strong, cylindrical stalks and recurrent roots close to its base (Basu and Arivukkarasu, 2006; Swain et al., 2008). It typically thrives in humid regions (Modi and Shah, 2017), and local tribes have uses the dried and pulverized plant to treat fever and cough. It has been attributed to vermifugal and diuretic properties (Khalid, 2010; Swain et al., 2008). Tinea capitis, a fungal scalp infection that often affects adolescents, is treated by applying freshly crushed leaf with castor oil to the scalp (Basu and Arivukkarasu, 2006; Nandha Kumar et al., 2019; Subramanian et al., 1969; Swain et al., 2008).

Although plant secondary metabolites have gained considerable attention in drug development, the active phytocompounds of *R. repens* have not been extensively explored. In addition, the literature has provided only limited insight into these phytochemicals and their evaluation through *in silico* methods (molecular docking and ADME), and their *in vitro* antioxidant, antibacterial, and cytotoxicity properties. The key objective of this study is to determine and assess the pharmacologically active compounds in *R. repens*. Antioxidant activity and cytotoxicity were evaluated using DPPH and MTT assays. Pharmacokinetic characteristics were studied using ADME to explore drug-likeness properties. Docking was performed for GC-MS identified compounds against the SarA protein of *Staphylococcus aureus*.

## 2 Experimental procedure

### 2.1 Plant sample collection and preparation

A plant sample collected from Amirthi village (Tamil Nadu, India) was identified and authenticated by the Botanical Survey of India (BSI-Coimbatore district, Tamil Nadu state, India) as *Rungia repens* (L.) Nees (No. BSI/SRC/5/23/2021/Tech/33). Its leaves were shade-dried, powdered, and then stored in an air-tight container. Extraction was carried out on 10 g of leaf with 100 mL of ethanol solvent. The crude extract was evaporated under vacuum to remove the solvent. The percentage yield of the dried sample was calculated by the following equation:

(%) yield = dried crude extract/amount of the dried leaves used *100 (Kalaichelvi and Dhivya, 2017; Keskin et al., 2024).

### 2.2 Phytochemical screening

*R. repens* was subjected to phytochemical screening as per Harborne (1998), analyzing the presence of phytochemicals such as terpenoids, alkaloids, tannins, saponins, flavonoids, and phenols (Harborne, 1998; Sharma and Veerachamy, 2013).

### 2.3 Ultraviolet-visible spectrophotometer analysis of *R. repens*

*R. repens* leaf extract was diluted using ethanol and was exposed to UV-visible spectrophotometric assessment. A Shimadzu UV-1800 analyzer was utilized to attain prominent peaks at the spectral range 200–800 nm (Kalaichelvi and Dhivya, 2017; Keskin et al., 2024; Rani et al., 2016).

### 2.4 Fourier transform infrared (FTIR) spectroscopy

FTIR was interpreted by scanning the samples at 4000 to 400 cm^-1^ wavelength using an FTIR spectrophotometer (Shimadzu, Japan, Thermo Fisher Scientific, USA). Exploring the different absorption bands perceived in the spectra led to the recognition of functional groups (Aguree et al., 2025; Talib and Aftab, 2021).

### 2.5 Folin–Ciocalteu assay for phenol quantification

The Folin–Ciocalteu method was employed to calculate the total phenol content in the crude sample with certain modifications. For the analysis, gallic acid standards of 20, 40, 60, 80, and 100 μg/mL and 100 μL of 1 mg/mL concentration of extracts were diluted to 500 μL with methanol and mixed with 2.5 mL of Folin–Ciocalteu (10%) solution, followed by incubation of 3 min at room temperature, then 2 mL of 2% sodium carbonate was added. After incubating at room temperature for 30 min, the absorbance at 760 nm was quantified. The TPC was estimated using milligram gallic acid equivalent/dry weight (gram) (El and Ahmed, 2025).

### 2.6 Quantification of flavonoids (TFC)

TFC was determined by adding 500 µL of sample aliquot and 500 µL of quercetin standard solution (20–120 μg/mL), and then 0.2 mL 10% potassium acetate (10%) solution was incubated for 5 minutes. Then, 0.2 mL of 10% AlCl_3_ was added. Using distilled H_2_O, the final volume was set to 5.0 mL and stirred. The outcomes were interpreted as quercetin equivalents (mg) per dry weight (100 g), with the measurement taken at 420 nm absorbance (Al Matani et al., 2015; Bharati et al., 2025; Shah and Hossain, 2014).

### 2.7 Assessment of radical-scavenging activity

A slightly modified 2,2-diphenyl-1-picrylhydrazyl (DPPH) radical-scavenging study was employed to evaluate the antioxidant properties of the extracts. Ascorbic acid was chosen as the standard, and crude extracts of 100, 200, 300, 400, 500 and 600 μg/mL concentration were prepared in DMSO. We added 1 mL of DPPH solution (25 μg/mL in ethanol) to 1 mL of each extract as well for standard. After agitation, the mixtures were incubated in the dark (37 °C) for 30 minutes. A reading at 517 nm was then taken. An increased activity of free radical scavenging was evidenced by a decrease in absorption. The following formula was employed to determine the percentage of the DPPH scavenging activity (Atere et al., 2018; Rajopadhey and Upadhey, 2013):

$$\text{Radical scavenging percentage}=\left(\frac{\text{CB}-\text{SB}}{\text{CB}}\right)\;X\;100,$$

Control Absorbance−CB,

Sample Absorbance−SB.

### 2.8 GC-MS chromatography

GC-MS analysis of the ethanol extract from *R. repens* leaf was performed using an Agilent GC 7890A/MS5975C instrument. Separation was achieved with an Agilent DB-5MS column with a specification of 30 m long, 0.25 mm internal diameter, and a thickness of 0.25 μm. Helium gas was employed at a rate flow at 1 mL/min. A 0.001 mL aliquot of the sample was injected using an auto-injector into the GC system paired with a mass spectrometer. The column oven operating temperature was 50 °C, which was raised 15 °C every minute until reaching 250 °C, where it remained for 5 minutes. The temperature was elevated to 315 °C, then maintained there for 16 min, at a rate of 20 °C per minute. The acquired spectra were compared to the NIST library to identify the compounds (Kim et al., 2017).

### 2.9 Cell viability assay

The mouse fibroblast L929 cell line procured from NCCS (National Centre for Cell Science, Pune, India) was seeded using a microtiter plate with a density of 1.0 × 10^4^ cells per well in a medium of Dulbecco’s modified-eagle medium, FBS (fetal bovine serum-10%) and antibiotic/antimycotic (1X) solution incubated at 37 °C in CO_2_ (5%) (Mahalakshmi and Parthasarathy, 2023). The wells were rinsed with 0.2 mL sterile phosphate buffer saline (1X concentration PBS), then the cells were treated with *R. repens* crude extract at various concentrations. Dimethyl sulfoxide (30%) was used as a reference control in serum-free medium and incubated 24 h. The medium was aspirated at the end of the treatment period. A 500 μg/mL quantity of 3- (4,5-dimethylthiazol-2-yl) 2,5-diphenyltetrazolium bromide reconstituted in PBS was supplemented and incubated at 37 °C for 4 h in a CO_2_ incubator. After incubation, the medium was aspirated and rinsed with 0.2 mL of sterile PBS. The formazan crystal was solubilized in 0.1 mL of DMSO, resulting in a purple blue color. The optical density was determined with a microplate spectrophotometer analyzer at 570 nm (Campoccia et al., 2021; Keerthika and Sandhya Raghu, 2021; Mahalakshmi and Parthasarathy, 2023; Prasathkumar et al., 2021; Sangeetha and Rajamani, 2018).

### 2.10 Antibacterial efficacy

Well diffusion, a previously developed technique, was used to evaluate the antibacterial properties of *R. repens* extracts against particular bacterial strains such as *P. aeruginosa* (MTCC 2582) and *S. aureus* (MTCC 3160). A consistent coating of bacterial suspension was applied over agar plates, which were left to air dry. Extract of 0.1 mL was added to wells that had been made in the agar. To assess antibacterial efficiency, the diameter of the zone of inhibition was measured after the plates had been incubated at 37 °C for 24 h (Alnaimat et al., 2012; Sharif et al., 2025).

### 2.11 Minimum inhibitory concentration of *R. repens*

Two-fold serial dilution was adopted to determine the minimum inhibitory concentration (MIC) of *R. repens*. In brief, 0.01 mL of bacterial suspension with a cell density of 1.5*10^8^ CFU/mL was dispensed in wells of Muller–Hinton broth (MHB). *R. repens* (2,000–62.5 μg/mL) was also added to the wells. *S. aureus* growth was compared with the control cultures by measuring the absorbance at 600 nm (Ganesh et al., 2022). The minimal concentration of the extracts that showed growth inhibition was marked “MIC.” The experiment was tested in three replicates (Alghamdi, 2023; Elbouzidi et al., 2024; Li, H. et al., 2021; Molla et al., 2010).

### 2.12 Molecular docking

The *Staphylococcus* accessory regulator A (SarA–PDB ID:2FNP) crystal structure of *S. aureus* was retrieved from the Protein Data Bank, and ligand structures were acquired from the PubChem database (Hosen et al., 2023; Selvaraj et al., 2021). Protein and ligand preparation were performed using AutoDock Vina version 1.1.2 (water molecule removed, hydrogen atom added, and Gasteiger and Kollman charges introduced), and the protein and ligand in PDBQT format were subsequently saved for docking analysis. The next step was to employ the grid box to cover the entire surface of the protein. Its dimensions of x = 16.598, y = −26.698, and z = −16.664 were configured. Binding affinities were calculated, and the receptor–ligand binding was revealed and displayed using BIOVIA Discovery Studio (Selvaraj et al., 2021).

### 2.13 ADME analysis

Pharmacokinetic analysis of the identified bioactive compounds was carried out using Swiss-ADME (http://www.Swisadme.ch). Significant ADME parameters were studied, such as permeability predictions using the boiled-egg model for GIT absorption and blood-brain barrier penetration (BBB), oral bioavailability (by means of Lipinski’s rule of five), and bioavailability radar parameters (polarity, size, solubility, lipophilicity, flexibility, and saturation). Furthermore, ADMET analysis computed polar surface area, protein binding, BBB permeability, CYP2D6 inhibition, solubility, absorption, and log P (Degfie et al., 2022; Elbouzidi et al., 2024; Lagorce et al., 2017; Vignesh et al., 2025).

### 2.14 Statistical interpretation

All experiments were performed in triplicate, and the values shown as Mean ± SD. ANOVA was conducted using *Graphpad Prism*. *P < 0.05* value differences were found to be significant.

## 3 Result and discussion

### 3.1 Yield of extract and qualitative analysis

The percentage yield of *R. repens* was determined to be 5% ± 0.50%. The sample extract was kept refrigerated (4 °C) for assays. Plant metabolite profiling showed the positive results for terpenoids, flavonoids, and phenols (Table 1). Swain et al. (2008) showed the existence of flavonoids, phenols, terpenoids, carbohydrates, and tannins. Thus, the extraction yield and the presence of phytoconstituents may vary with factors such as place of origin, environmental conditions, choice of solvent used for extraction, and certain plant parts used, as explained by Barbouchi et al. (2024).

**TABLE 1:** Phytochemical screening of *R. repens* leaf extract.

Phytochemical	*R. repens*
Phenol	+
Tannin	+
Alkaloid	-
Flavonoid	+
Carbohydrate	+
Terpenoids	+
Fixed oil and fat	-
Proteins	-
Saponin	-
Positive (+), negative (−)	

### 3.2 *R. repens* UV-visible spectra

**FIGURE 1:**
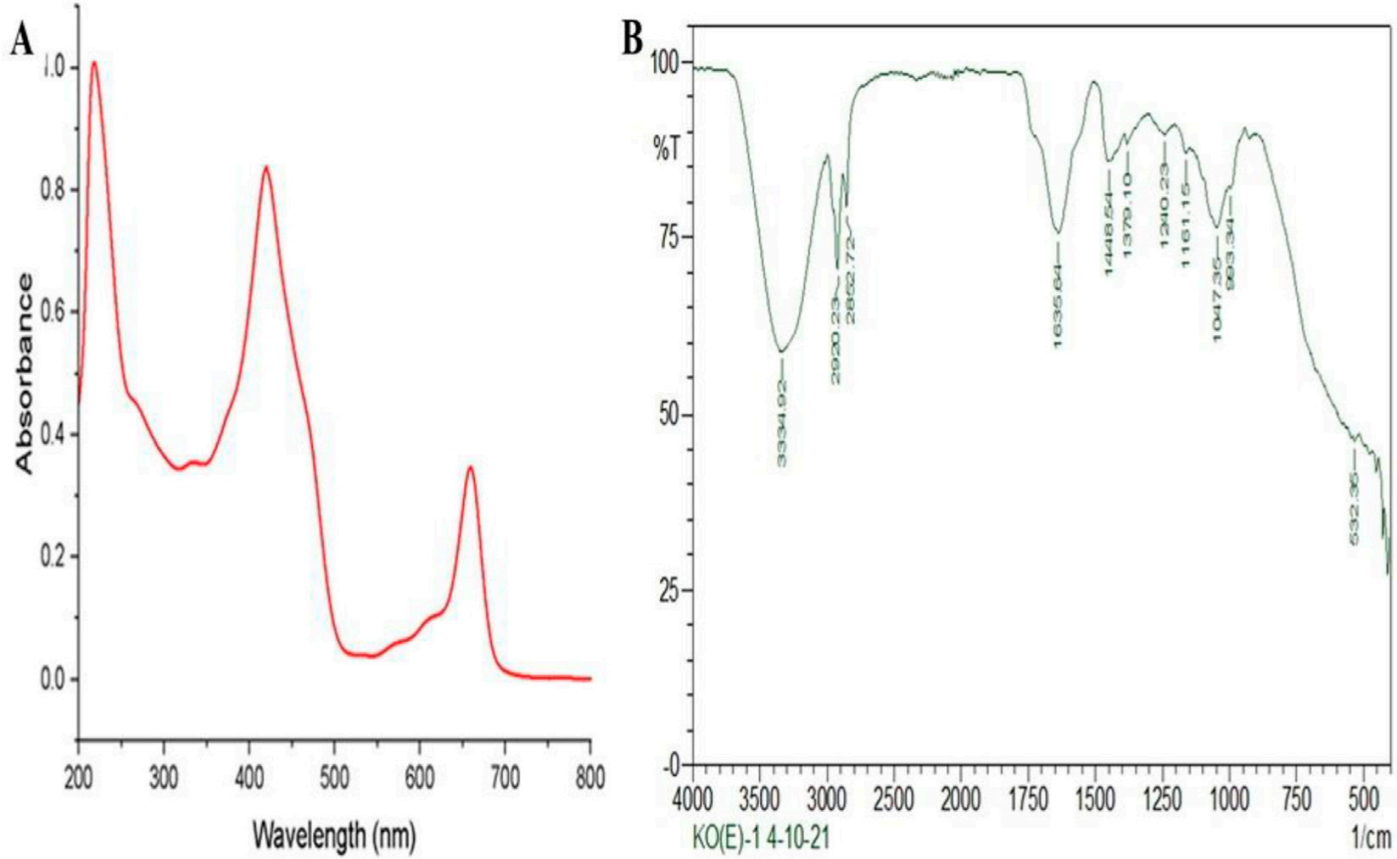
**(A)** UV-visible absorption spectrum of *R. repens*. **(B)** FTIR of *R. repens* ethanol extract.

The UV visible spectra for *R. repens* leaf extract were observed to recognize molecules containing π-bonds, σ-bonds, aromatic rings, lone pair electrons, and chromophoric groups in the UV–Vis range (200–800 nm) of the electromagnetic spectrum (Kalaichelvi and Dhivya, 2017). In this study, the ethanol leaf extract showed an absorption peak at 217, 270, 333, 417, and 660, respectively (Figure 1A) (Keskin et al., 2024; Kumar and Pandey, 2013; Patle et al., 2020; Song et al., 2020).

### 3.3 FTIR spectral interpretation of the leaf extract

The infrared spectrum of *R. repens* was obtained in the bandwidth of 4000–500 cm^−1^ to identify the chemical groups and molecular linkages of the active compounds based on the characteristic absorption peaks corresponding to the characteristic region (Figure 1B) (Keskin et al., 2024; Singh et al., 2022). FTIR analysis showed the presence of major peaks at 3334.92 cm^−1^, 2902.23 cm^−1^, 2852.72 cm^−1^, 1635.64 cm^−1^, 1448.54 cm^−1^, 1379.10 cm^−1^, 1240.23 cm^−1^, 1161.15 cm^−1^, 1047.35 cm^−1^, 993.34 cm^−1^, and 532.35 cm^−1^. The band at 3334.2 cm^−1^ indicated the presence O-H stretching, indicating phenolic compounds, which are responsible for their antioxidant activities. Band at 2902.23 cm^−1^ indicated C-H stretching of alkanes, and the wavenumber 2850.72 cm^−1^ corresponded to O-H stretching. The band at 1635.64 cm^−1^ is possibly assigned to the C=C aromatic ring, flavonoids, and the stretching frequency of C-C bonds. The characteristic band at 1448.54 cm^−1^ corresponds to the C-H bend, indicating the presence of alkene groups. The O-H bend peak was observed at 1379.10 cm^−1^. The band C-O stretching vibration was observed at 1240.23 cm^−1^, 1161.5 cm^−1^, and 1047.35 cm^−1^ (Figure 1B) (Agatonovic et al., 2021; Dauda et al., 2026; Jesse, 2020; Prasathkumar et al., 2021).

### 3.4 Quantification of phenol and flavonoid content

**FIGURE 2:**
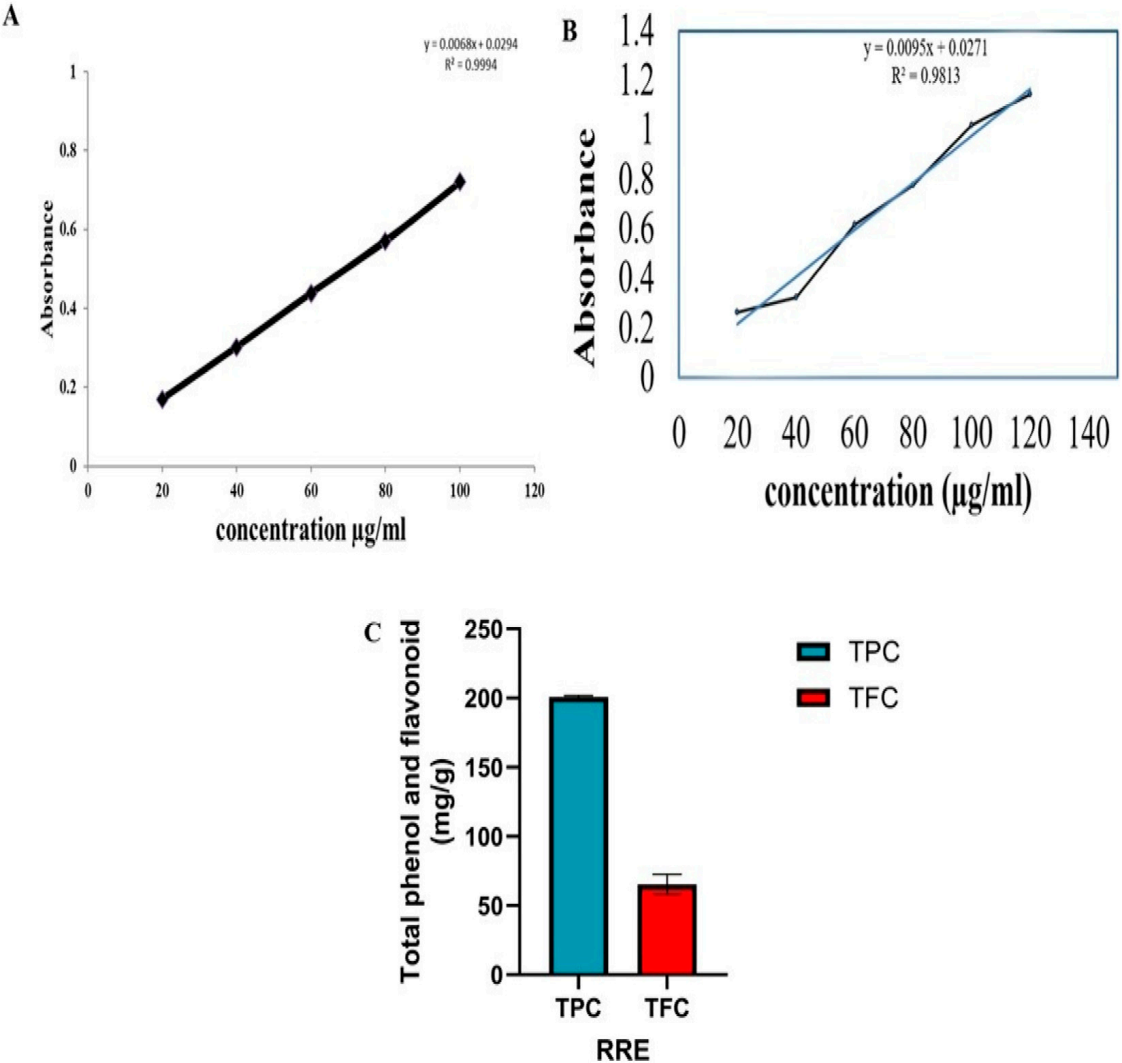
**(A)** Standard calibration curve of gallic acid for total phenol content. **(B)** Total calibration curve of quercetin standard. **(C)** TPC and TFC of *R. repens* ethanol extract.

Quantified phenol content was expressed as mg gallic acid equivalent (GAE)/g of extract using a standard calibration curve (Y = 0.0068x+0.0294, *R* ^2^ = 0.9994), which was identified as 202.40 ± 2.97 mg/g (Figures 2A,C). Similarly, flavonoid content was calculated using a quercetin calibration plot (Y = 0.0095x+0.0271, *R* ^2^ = 0.9813), which was determined to be 65.31 ± 7.2 mg QUE/g (Figures 2B,C). Phenolic compounds, which have ability to impede oxidative stress by scavenging free radicals and ROS, might be responsible for the antioxidant property, which promotes resistance against oxidative stress-related diseases. It is also known to have anti-inflammatory and antibacterial activity (Shi L. et al., 2022).

### 3.5 Antioxidant activity

**FIGURE 3:**
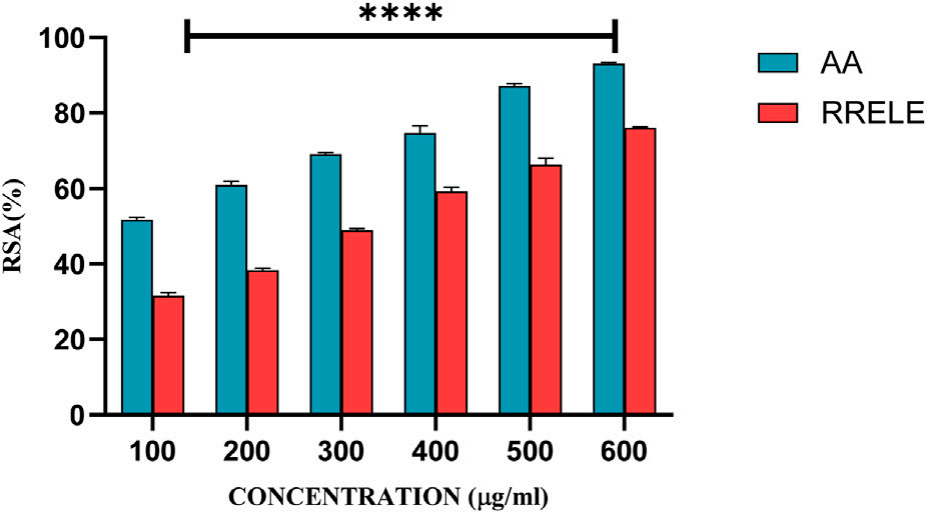
DPPH free radical-scavenging activity of RRELE (*R. repens)* compared with ascorbic acid (AA) (Mean ± SD, n = 3).

Table 2 and Figure 3 summarize the free radical-scavenging activity of *R. repens*. Crude-extract-free radical activity was determined to be 31.6%, 38.409%, 48.972%, 59.27%, 66.37%, and 76.16% at various concentrations of 100 μg/mL, 200 μg/mL, 300 μg/mL, 400 μg/mL, 500 μg/mL, and 600 μg/mL of *R. repens* leaf extract (Figure 3). The antioxidant capacity of standard ascorbic acid was 51.8%, 60.7%, 69.1%, 80.7%, 90.8%, and 93.1% at 100 μg/mL, 200 μg/mL, 300 μg/mL, 400 μg/mL, 500 μg/mL, and 600 μg/mL, respectively (Figure 3). The IC_50_ of *R. repens* was 311.9 ± 1.27 compared to that of ascorbic acid 75.08 ± 4.28. The results accord with previous research on the antioxidant potential of *R. repens* plant (Nandha Kumar et al., 2019). The presence of flavonoids, belonging to a polyphenol class, exhibits antioxidant activity by a free-radical-scavenging mechanism (Kumar and Pandey, 2013).

**TABLE 2:** *R. repens* leaf extract antioxidant activity.

Antioxidant activity	Standard	*R. repens*
% RSA (600 μg/mL)	93.1 ± 0.3	76.166 ± 0.15
IC_50_ (µg/mL)	75.081 ± 4.28	311.9 ± 1.27

### 3.6 Identification of volatile compounds

**FIGURE 4:**
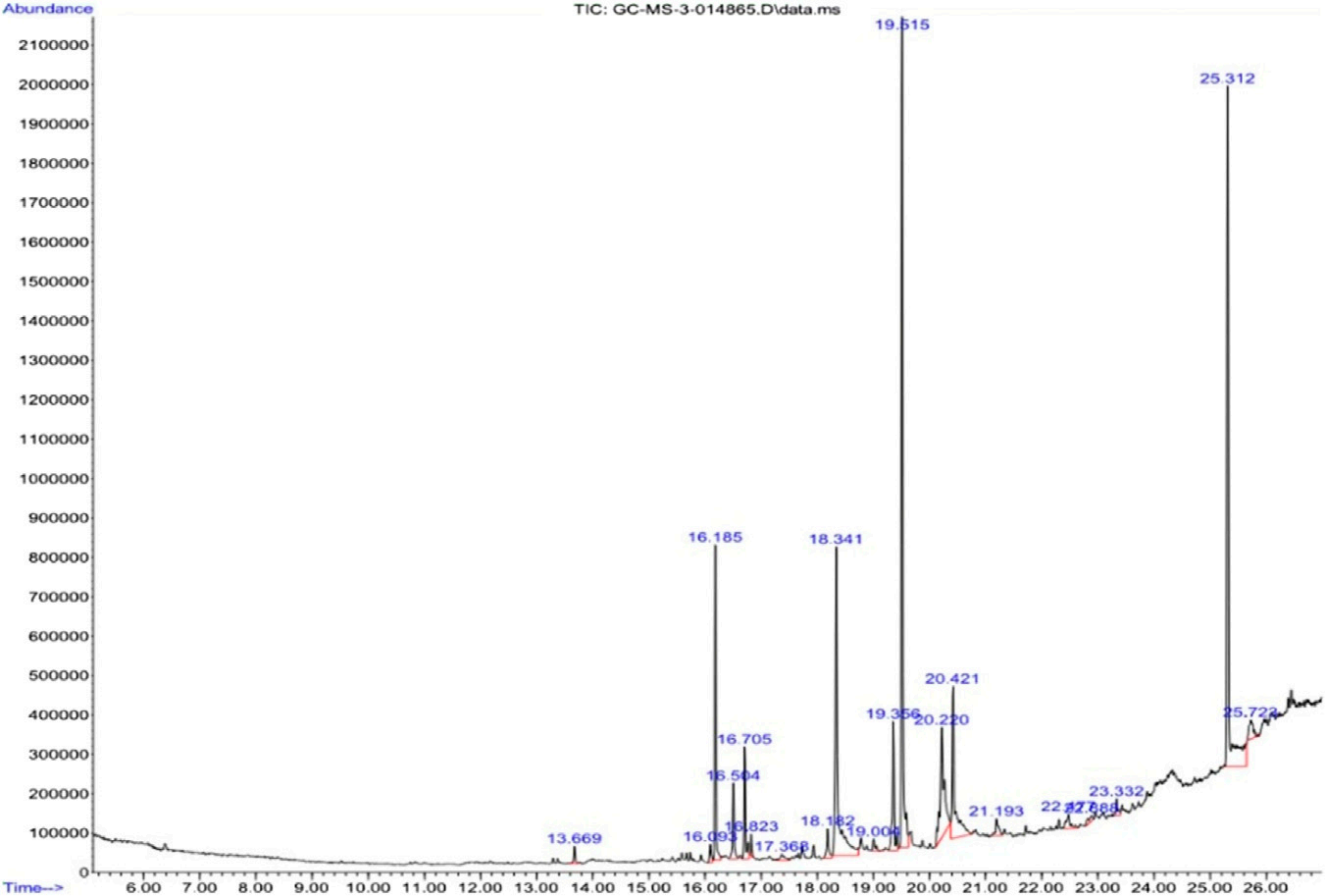
Chromatogram of the *R. repens* (ethanol) extract.

GC-MS profiling tentatively identified 19 compounds in the *R. repens* ethanol extract (Table 3; Figure 4). The mass spectra for the identified compounds are given in Supplementary Figure S1. The primary components were identified as phytol, squalene, octadecanoic acid, phthalic acid, butyl undecyl ester, 9,12,15-octadecatrienoic acid ethyl ester (Z,Z,Z), and 2,6,6-trimethylbicyclo [3.1.1]heptane. In addition, 1-hexadecyne, cyclohexanol, 1-ethynyl, and stigmasta-3,5-dien-7-one were the next most prevailing bioactive compounds (Figure 4; Table 3). Compounds identified via GC-MS analysis have been studied in the literature to demonstrate various biological effects, including anticancer, antimicrobial, antioxidant, and anti-inflammatory properties (Nishanthini et al., 2014, Othman et al., 2015; Rizvi et al., 2023).

**TABLE 3:** Volatile compounds identified in ethanol extract of *R. repens* leaf by GC-MS.

Retention time (minutes)	Compound name	Molecular weight	Molecular formula	Area %
13.669	Phenol, 2,4-bis(1,1-dimethylethyl)	206.32 g/mol	C_14_H_22_O	0.53
16.093	2-Hexadecene,3,7,11,14-tetramethyl-, [R-[R*R*-(E)]]-	280.5 g/mol	C_20_H_40_	0.57
16.185	2,6,6-trimethylbicyclo [3.1.1]heptane, (1.alpha.,2.beta.,5.alpha.)-	138.25 g/mol	C_10_H_18_	7.42
16.504	1-Hexadecyne	222.41 g/mol	C_16_H_30_	2.13
16.705	Cyclohexanol, 1-ethynyl	124.18 g/mol	C_8_H_12_O	3.14
16.823	2-Pentadecanone, 6,10,14-trimethyl-	268.5 g/mol	C_18_H_36_O	0.50
17.368	Bicyclo [2.2.1]heptane, 2,2,3-trimethyl	138.24 g/mol	C_10_H_18_	0.54
18.182	Acetic acid, 10,11-dihydroxy-3,7,11,-trimethyldodeca-2,6-denyl ester	298.4 g/mol	C_17_H_30_O_4_	1.21
18.341	Phthalic acid, butyl undecyl ester	376.5 g/mol	C_23_H_36_O_4_	14
19.004	N-[2-(Dimethylamino)ethyl]-2-phenyl-4-quinolinamine	159.27 g/mol	C_23_H_36_O_4_	0.54
19.515	Phytol	296.5 g/mol	C_20_H_40_O	22.64
20.220	Octadecanoic acid	284.5 g/mol	C_18_H_36_O_2_	8.20
20.421	9,12,15-Octadecatrienoic acid, ethyl ester, (Z,Z,Z)-	306.5 g/mol	C_27_H_34_O_2_	7.46
21.193	9-Decen-2-one	154.25 g/mol	C_10_H_18_O	0.95
22.477	1-Hentetracontanol	593.1 g/mol	C_41_H_84_O	1.09
22.888	7-Chloro-1-[[3-(dimethylamino)propyl]imino]-1,3,4,10-tetrahydro-10-hydroxy-3-(2-methoxyphenyl)-9(2H)-acridinone	454 g/mol	C_25_H_28_ClN_3_O_3_	0.54
23.332	3-Trifluoroacetoxypentadecane	324.4 g/mol	C_17_H_31_F_3_O_2_	0.65
25.312	Squalene	410.7 g/mol	C_30_H_50_	22.23
25.723	Stigmasta-3,5-dien-7-one	410.7 g/mol	C_29_H_46_O	1.45

### 3.7 Cytotoxicity prediction using the L929 cell line

**FIGURE 5:**
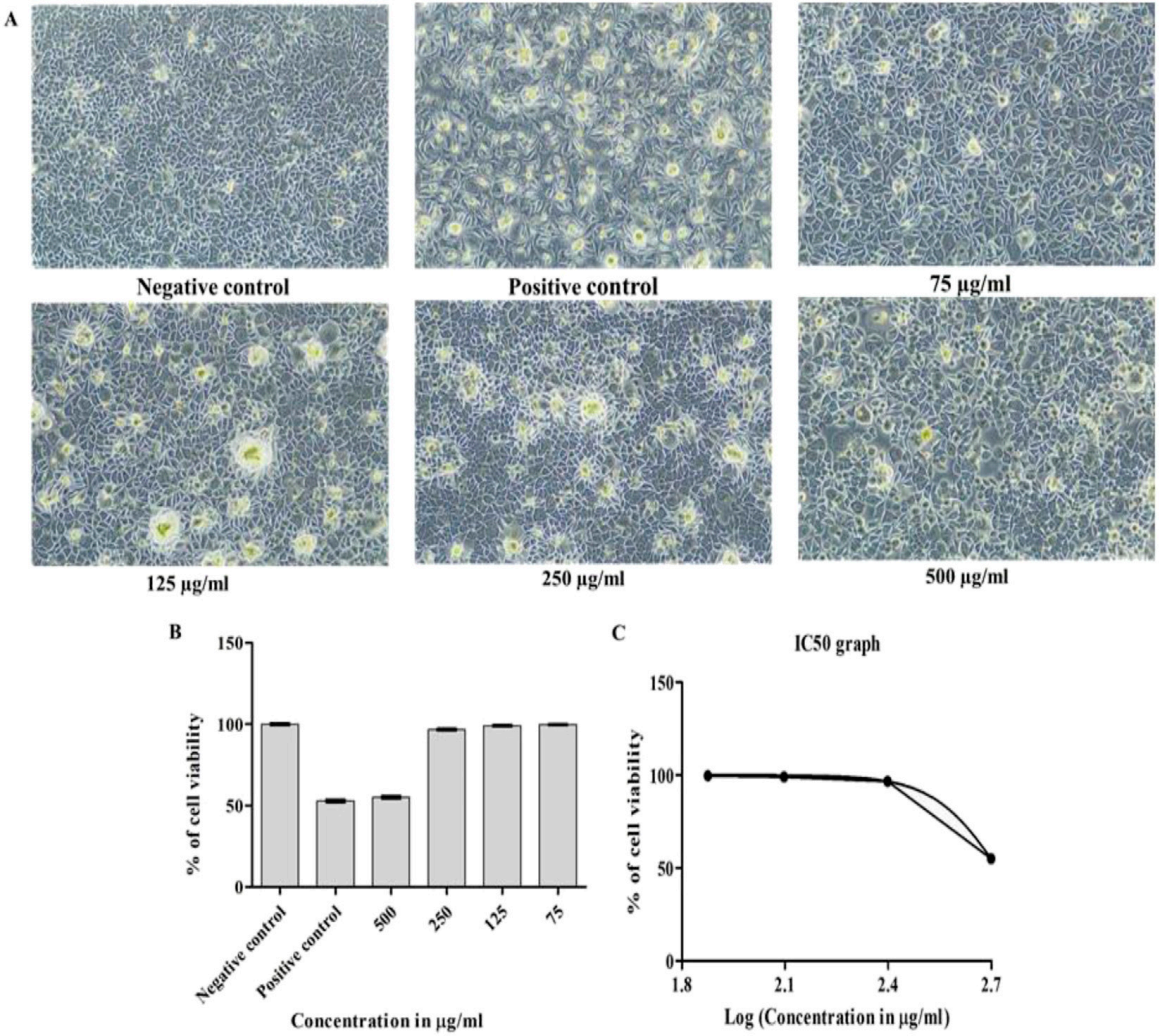
**(A)** Cytotoxicity of ethanol leaf extract of *R. repens* against L929 cell line with different concentration. **(B)** Graph displaying percentage viability versus concentration of *R. repens* extract and **(C)** IC_50_ graph.

No prior exploration was found to reveal the potency of *R. repens* on the proliferation of mouse fibroblast L929 cells. The concentrations 75, 125, 250, and 500 μg/mL were selected based on the preliminary screening that enabled the assessment of both cytotoxic and non-toxic effects of the extract. The L929 cell line was treated by *R. repens* following 24-h exposure to the extract, and morphological alterations were observed (Figure 5A). Lower concentrations (75, 150, and 250 μg/mL) showed rounded, enlarged cells that exhibited intracellular vacuole-like structures with 96.68%, 99.08%, and 99.66% cell viability (Figure 5B), with an IC_50_ value graph illustrated in Figure 5C. Cells treated with 500 μg/mL *R. repens* extract, however, formed dense aggregates, exhibiting irregular, rounded morphology and substantially suppressed cell proliferation at 55.10%. The experimental positive and negative control treated cells showed 21% and 100% cell survival, respectively. Taken together, the results indicate that the extract indicates concentration-dependent cytotoxicity and is biocompatible at lower concentrations (75,125, and, 250 μg/mL).

### 3.8 Antibacterial efficacy

*R. repens* bacterial inhibition was measured by a zone of inhibition (mm) (Table 4). It exhibited inhibition against *S. aureus*, whereas no inhibitory effect was observed in *P. aeruginosa*. The outer membrane of *P. aeruginosa* consists of an asymmetric bilayer of phospholipids and lipopolysaccharides (LPS) in porins that form β-barrel protein channels. It might serve as a selective barrier limiting the penetration of metabolites and contribute to resistance (Delcour, 2009; Omokhua et al., 2017). It may also be due to diverse aspects, like the biogeographical variation of the plant, discrepancies in extraction strategy, phenological stages, and solvent preference (Asfaw et al., 2023, Mostafa et al., 2018).

**TABLE 4:** Antibacterial activity exhibited by *R. repens*.

Microorganism	Zone of inhibition (mm)	
	*R. repens*	Tetracycline
*S. aureus (MTCC 3160)*	14 ± 1.73	29 ± 1.52 (Tetracycline)
*P. aeruginosa (*MTCC1688*)*	-	30.0 ± 0.58

Experiments were done in triplicate, and data are expressed as mean ± SD, values (diameter of the well 6 mm).

### 3.9 Minimum inhibitory concentration (MIC)

The MIC of *R. repens* toward *S. aureus* was determined to be 1.0 mg/mL. Results revealed that extracts with minimum inhibitory concentration work effectively against *S. aureus.*

### 3.10 Molecular docking of SarA

**FIGURE 6:**
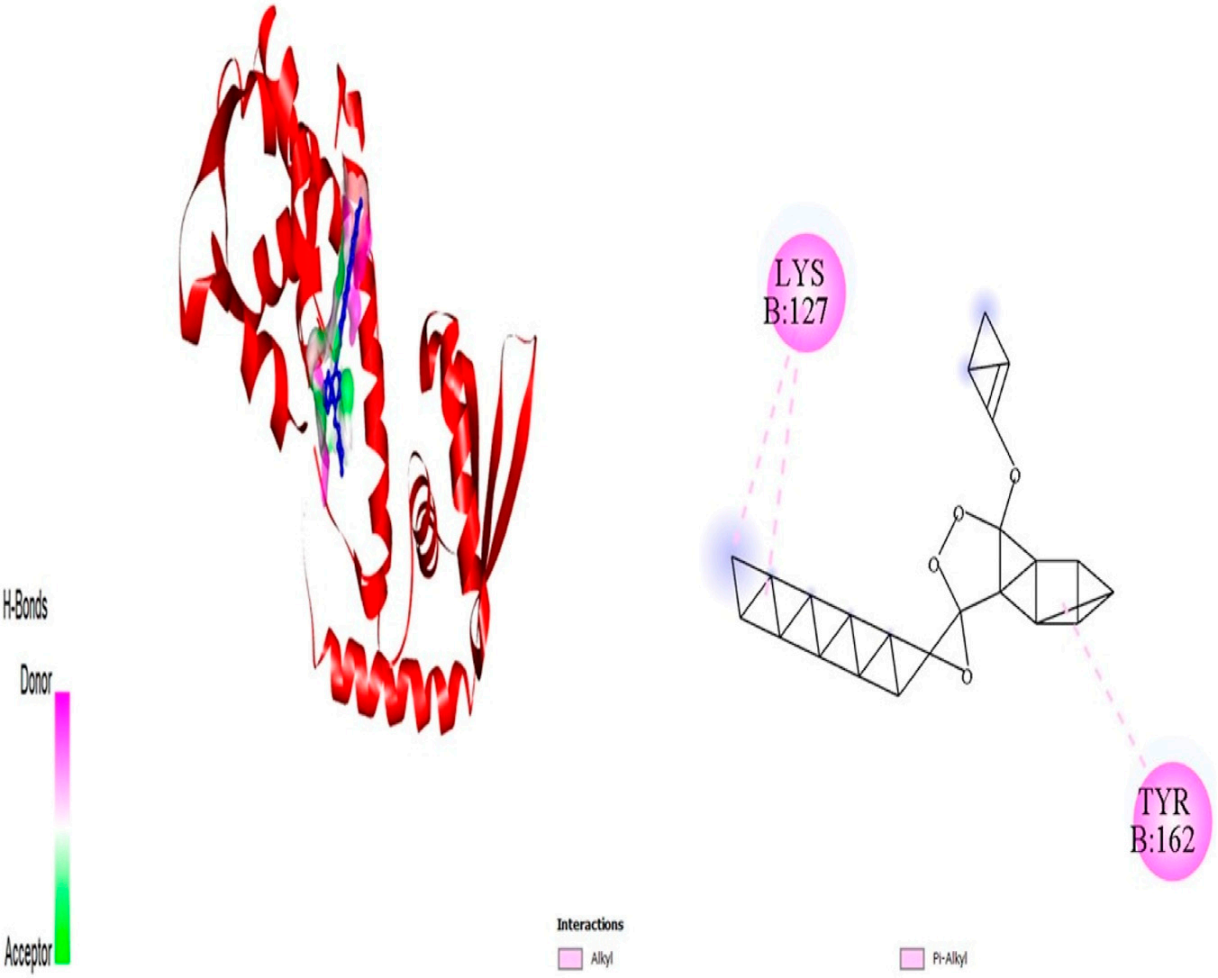
2D and 3D interaction plot of phthalic acid butyl undecyl ester with optimal docking scores into the active site of SarA.

Staphylococcal accessory regulator A (SarA) was selected as a molecular docking target owing to its essential role in controlling *S. aureus* pathogenicity. Key aspects of bacterial pathogenicity, such as adhesion, biofilm formation, and toxin generation, are influenced by SarA. Targeting SarA decreases the ability of resistance development via an anti-virulence approach contrary to conventional antibiotics (Dorcheh et al., 2022; Liu et al., 2024). Therefore, docking analysis was performed for the compounds in the ethanol leaf extract of *R. repens* with SarA of *S. aureus*. The docking scores and distance and type of interaction, category, and the number of hydrogen bonds formed of the compounds with SarA are given in Supplementary Table S1 and Supplementary Figure S2. Figure 6 illustrate 2D and 3D interactions of the best docked compound. The 2D and 3D structure and binding affinity of the bioactive compounds *R. repens* are given in the Supplementary Figure S2.

The compound’s phthalic acid butyl undecyl ester displayed the highest binding efficiency of approximately −10.1 kcal/mol (Figure 6). The binding affinity of all the compounds are given in Supplementary Table S1. However, phthalic acid butyl undecyl ester did not form any hydrogen bond with the amino acid residues. These findings suggest that *R. repens* demonstrated stronger binding affinity. Nevertheless, there was no hydrogen bond formed between phthalic acid butyl undecyl ester and the amino acid residues of the enzyme activity. These findings deserve further experimental validation as they rely on an *in silico* approach.

### 3.11 ADME properties

Assessing pharmacokinetic characteristics with Swiss ADME (http://www.swissadme.ch) is indispensable for forecasting how a drug will perform within the human body (Al-Samawi et al., 2025; Vijay et al., 2025). Understanding these properties is a key factor in determining the success of any potential bioactive compound. *R. repens* secondary metabolite identified by GC-MS chromatography was evaluated using the Swiss ADME website in accordance with significant physicochemical characteristics involving polarity, solubility, lipophilicity, carbon saturation, and molecular flexibility, which are shown by the number of rotatable bonds, to determine their drug likeness (Supplementary Tables S2–S6) (Afroz Shoily et al., 2025).

**FIGURE 7:**
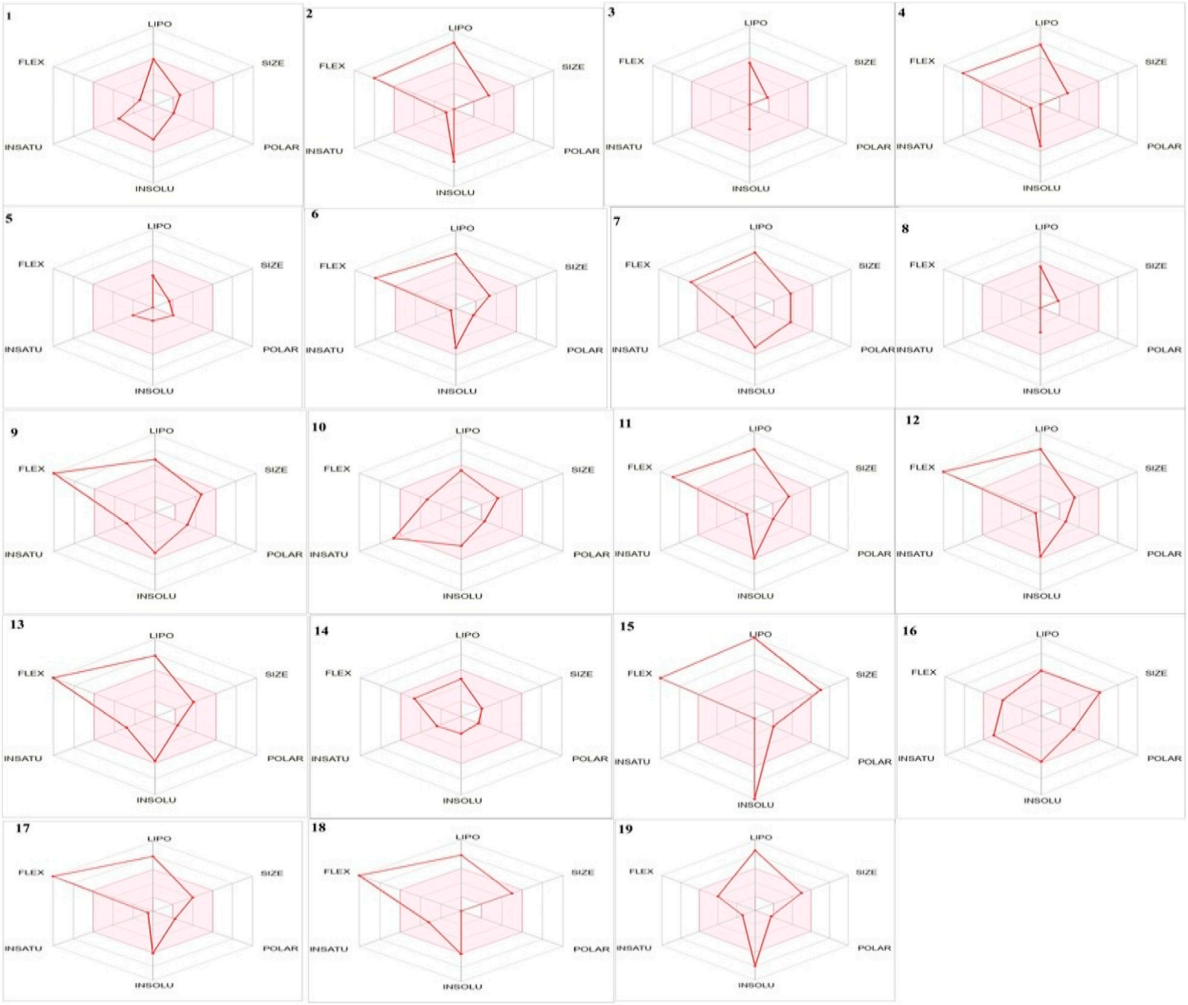
**C1**-phenol,2,4-bis(1,1-dimethylethyl), **C2**-2-hexadecene,3,7,11,14-tetramethyl-, [R-[R*R*-(E)]]-, **C3**-2,6,6-trimethylbicyclo [3.1.1]heptane, **C4**-1-hexadecyne, **C5**-cyclohexanol,1-ethylnyl, **C6**-2-pentadecanone,6,10,14,trimethyl-, **C7**-bicyclo [2.2.1]heptane, 2,2,3-trimethyl, **C8**-acetic acid,10,11-dihydroxy 3,7,11,-trimethyldodeca-2,6-denyl ester, **C9**-phthalic acid, butyl undecyl ester, **C10**-N-2 [2-(dimethylamino)ethyl]-2-phenyl-4-quinolinamine, **C11**-phytol, **C12**-octadecanoic acid, **C13**-9,12,15-octadecatrienoic acid, ethyl ester, (Z,Z,Z)-, **C14**-9-decen-1one, **C15**-1-hentetracontanol, **C16**-7-chloro-1-[[3-(dimethylamino)propyl]imino]-1,3,4,10-tertahydro-10-hydroxy-3-(2-methoxyphenyl)-9(2H)-acridion, **C17**-trifluoroacetoxypentadecane, **C18**-squalene, **C19**-stigmasta-3,5,-dien-7-one.

Lipinski’s and Veber’s rules were employed to analyze the physicochemical profiles that specify the ADME (absorption, distribution, metabolism, and excretion) traits of the bioactive compounds. Lipinski’s rule, a basic framework for determining compounds with drug-like traits, aids in distinguishing between those that might possess therapeutic value and those less likely to work. The five key variables considered by the rule are molar refractivity, log P (a measure of lipophilicity), number of H (hydrogen)-bond donor, number of H-bond acceptors, and molecular weight. Therefore, a compound is regarded as drug-like if it adheres to more than one of these conditions, such as the sum of the TPSA (polar surface area) of the compound, lipophilicity, P-glycoprotein (permeability-gp) substrate, gastrointestinal (GI) drug intake, solubility, BBB penetration (BBB-P), skin permeation, medicinal chemistry, and drug likeness (Supplementary Table S2). The bioavailability radar of the compounds of *R. repens* are given Figure 7. The pink portion of the radar denotes the appropriate range that determine drug-likeness properties (Supplementary Tables S2–S6) (Al samawi et al., 2025; Sabiri et al., 2025; Vijay et al., 2025).

The p-glycoprotein serves as crucial efflux pump since it delivers bioactive metabolites from extracellular, via the human intestinal lumen, to urine excretion and the peripheral nervous system (Al-Samawi et al., 2025; Alreshidi et al., 2020). Compounds such as 2-hexadecene, 3,7,11,14-tetramethyl-[R-R*R*-E]]-2-pentadecanone, 6,10,14-trimethyl, acetic acid, phytol, 1-hentetracontanol, 10,11- dihydroxy- 3,7,11-trimethyl-dodeca-2,6-dienyl ester, and 7-chloro-1-[[3-(dimethylamino) propyl] imino]-1,3,4,10-tetrahydro-10-hydroxy-3-(2-methoxyphenyl)-9(2H)-acridinone are substrates for permeability-gp. Others were predicted as non-permeability-gp substrates (Supplementary Table S3).

A number of compounds showed inhibitory outcomes on the cytochrome P450 enzymes, implying a potential for drug interactions. In particular: CYP1A2 was hindered by 1-hexadecyne and octadecanoic acid; CYP2C9 was hindered by phytol, squalene, and other drugs; CYP3A4 was inhibited by phthalic acid butyl undecyl ester and other similar compounds. Phenol, 2,5-bis(1,1-dimethylethyl) was shown to exhibit broad-spectrum inhibition of CYP2C19, CYP2C9, CYP2D6, and CYP3A4. In addition, CYP2D6 was suppressed and directed to inimical pharmacokinetic interactions (Supplementary Table S3) (Afroz Shoily et al., 2025; Foudah et al., 2022).

**FIGURE 8:**
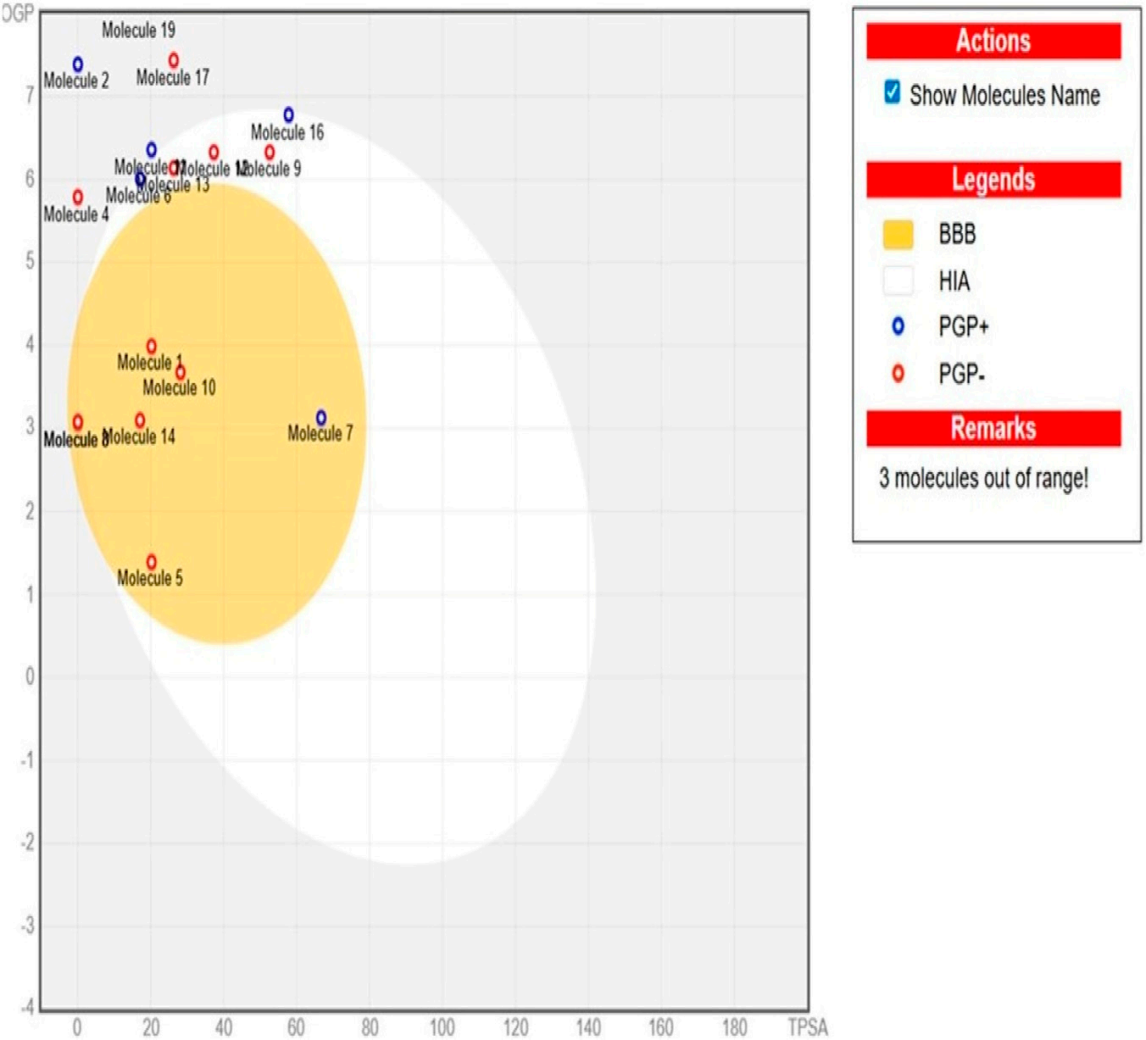
Boiled egg pictorial representation of bioactive compounds of *R. repens*.

The boiled-Egg assessment’s insights highlight the relevance of physical and chemical characteristics in the early stages of drug development. In this approach, the drug’s permeation via the eggshell and its dispersion inside the egg are assessed using a boiled egg as a proxy for the human body. The outcome of the molecules generated in a boiled egg projection is presented in Figure 8, wherein the egg white corresponds to human gastrointestinal absorption (HIA)-favorable compounds and the egg yolk to both the BBB and HIA molecules (Daina et al., 2017; Sen and Karati, 2026).

The boiled-Egg structure (Figure 8) conveys the possibility of *R. repens* secondary metabolites penetrating the BBB and their oral bioavailability. The graphic illustration shows that six compounds are inside the central region, pointing to a high plausibility of BBB penetration and significant oral absorption, while other compounds fall within the outermost region, offering restrained features. Compounds 1, 5, 7, 8, 10, and 14 demonstrate a proper evaluation of their drug-likeness properties in Figure 8. Appropriate TPSA and lipophilicity are both crucial for brain penetration, and oral absorption serves as the rationale for the prediction. These characteristics are pivotal for the pharmacokinetic trait of the drugs (Alhawarri et al., 2024; Sweety et al., 2025).

The log P_o_/_p_ (octanol-water partition coefficient) is a typical assessment of a compound’s lipophilicity, reflecting how it distributes itself between a lipid-like (octanol) and aqueous (water) phase. This property is crucial because it correlates with a molecule’s ability to cross biological membranes, which are largely composed of lipids. Compounds with low lipophilicity indicate that they are highly hydrophilic and have poor membrane permeability, since they cannot easily diffuse through the lipid bilayer of cell membranes (Batool et al., 2024). A consensus log P_o_/_p_ value was acquired by averaging lipophilicity predictions from the iLOGP, XLOGP3, WLOGP, MLOGP, and SILICOS-IT computational models (Daina et al., 2017; Ononamadu and Ibrahim, 2021). Drug likeness is appraised by bioavailability radar using six essential features: size, polarity, solubility, flexibility, saturation, and lipophilicity. Plotting within the ideal pink area indicates that a chemical is drug-like. The majority of the assessed compounds showed ideal values for each of the six essential features, indicating favorable oral bioavailability. These compounds included phenol 2,5-bis(1,1-dimethylethyl), 2,6,6-trimethylbicyclo [3.1.1]heptane. Some derivatives, however, exhibited significant lipophilicity (XLOGP3 > 5), which is linked to problems such as limited solubility, poor absorption, *in vivo* toxicity, and decreased receptor selectivity *in vitro*. Because of their poor pharmacokinetic and safety properties, substances with elevated lipophilicity are frequently not appropriate as medication candidates (Supplementary Table S4).

Bioactive metabolites must cross the BBB to target the central nervous system. Compounds like C2, C4, C6, C7, C9, C11, C12, C13, C15, C16, C17, C18, and C19 failed to cross the BBB and might have unfavorable effects in the central nervous system (CNS), but diverse bioactive metabolites have a likelihood of permeating the BBB (Figure 8). A bioavailability score for all the compounds is 0.55–0.85, indicating a chance of being bioavailable, except for 1-hentetracontanol, and 7-chloro-1-[[3-(dimethylamino)propyl]imino]-1,3,4,10-tetrahydro-10-hydroxy-3-(2-methoxyphenyl)-9(2H)-acridinone, which has 0.17. The percentage of the drugs that get into the blood circulation is a pivotal aspect of drug development. Gastrointestinal absorption is the foremost contributor to a drug’s bioavailability. The aqueous solubility of orally administered drug plays an important role in bioavailability (Supplementary Table S5) (Afroz Shoily et al., 2025; Sweety et al., 2025).

Instead of directly interacting with the intended biological targets, some chemical compounds and structural groups termed “PAINS” are known to interfere with biological assays by changing the assay results. Some of these substances have chemical characteristics that allow them to interact with DNA or proteins, leading to negative outcomes such as liver toxicity, CYP450 inhibition, or cancer. The underlying chemical reactivity of the chemicals themselves is often linked to these adverse effects (Supplementary Table S6) (Batool et al., 2024).

The compounds were screened for BRENK and PAINS alerts, with none exhibiting PAINS-related structural flags. This absence discloses the nonexistence of promiscuous structural motifs related to false-positive consequences in computational assays (Baell and Holloway, 2010). BRENK alerts pinpoint chemical fragments associated with probable toxicity, reactivity, or metabolic fluctuation. Compounds including 1-hexadecyne, cyclohexanol, phthalic acid butyl undecyl ester, phytol, and 9-decen-2-one showed one BRENK alert. Compound 7 depicted two alerts. While the importance of these alerts depends on the precise structural moiety involved, they deserve thorough evaluation during drug-likeness sorting to appraise uncertainties (Supplementary Table S6) (Ononamadu and Ibrahim, 2021).

## 4 Conclusion

*R. repens* is an ethno-pharmacologically significant plant. A GC-MS profile clarified the phytochemical profile of the plant, which is known to have wide pharmaceutical benefits. Its crude extract exhibited high total phenol and considerable flavonoid content, which might be responsible for radical-scavenging activity, further confirming their increased bioactivity. The present research validates the antibacterial potential of *R. repens*. The plant-derived compounds phthalic acid butyl undecyl ester showed binding affinity against the SarA of *S. aureus*, via docking analysis. ADME assessment showed favorable physiochemical characteristics like drug likeness, bioavailability, and toxicity and their potential as favorable drug candidates. An *in vitro* cytotoxicity study of *R. repens* against the *L929* cell line exhibited a non-toxic effect at lower concentrations. However, further *in vitro* and *in vivo* investigations are essential to elucidate the precise mechanisms of action of its bioactive phytochemical compounds.

## Data Availability

The original contributions presented in the study are included in the article/Supplementary Material; further inquiries can be directed to the corresponding author.
