# Supplementary material for: GC-MS profiling, in vitro antioxidant and toxicity evaluation, and in silico pharmacokinetic and molecular docking prediction of the phytocompounds of Rungia repens (L.) Nees targeting SarA of Staphylococcus aureus
Source: Front Chem. 2026 Jul 22;14:1778720. doi: 10.3389/fchem.2026.1778720 (PMC13438309; doi:10.3389/fchem.2026.1778720)
Supplement: Supplementary file 1 [file DataSheet1.docx]

# GC-MS profiling, *in vitro* antioxidant and toxicity evaluation and *in silico* pharmacokinetic and molecular docking prediction on phytocompounds of *Rungia repens(L.) Nees* targeting SarA of *Staphylococcus aureus*

Vaidagi balaji**^1^**, Gayathri Mahalingam**^1^***

**^1^** Department of Bio-medical Sciences, School of Bio-Sciences and Technology (SBST),

Vellore Institute of Technology (VIT), Vellore – 632 014, Tamil Nadu, India.

*Corresponding author: Gayathri Mahalingam

E-Mail: [gayathrigopinath@vit.ac](mailto:gayathrigopinath@vit.ac)

**Figure S1**. Mass spectrum of ethanol extract of *R. repens* leaf constituents (Peak # 1 to 19) as identified by GC-MS.

**Fig S2. 2D and 3D structure of the compounds of the R. repens leaf extract**

**Table S1. Binding affinity of docked ethanol extract of *R. repens* with SarA**

**Table. S2 Physiochemical characteristics traits of *R. repens***

**Table. S3 Pharmacokinetics properties**

| *R. repens* compounds | No. of | No. of HBA | No. of HBD | MR | TPSA |
| --- | --- | --- | --- | --- | --- |
|  | Rot |  |  |  |  |
| Phenol, 2,4-bis(1,1-dimethylethyl) | 2 | 1 | 1 | 67 | 20.23 |
| 2-Hexadecene,3,7,11,14-tetramethyl-,[R-[R*R*-(E)]]- | 12 | 0 | 0 | 97.78 | 0 |
| 2,6,6-trimethylbicyclo[3.1.1]heptane, (1.alpha.,2.beta.,5.alpha.)- | 0 | 0 | 0 | 45.7 | 0 |
| 1-Hexadecyne | 12 | 0 | 0 | 77.19 | 0 |
| Cyclohexanol, 1-ethynyl | 0 | 1 | 1 | 37.82 | 20.23 |
| 2-Pentadecanone, 6,10,14-trimethyl- | 12 | 1 | 0 | 88.84 | 17.07 |
| Bicyclo[2.2.1]heptane, 2,2,3-trimethyl | 10 | 4 | 2 | 86.53 | 66.76 |
| Acetic acid, 10,11-dihydroxy-3,7,11,-trimethyldodeca-2,6-denyl ester | 0 | 0 | 0 | 45.7 | 0 |
| Phthalic acid, butyl undecyl ester | 17 | 4 | 0 | 111.49 | 52.6 |
| N-[2-(Dimethylamino)ethyl]-2-phenyl-4-quinolinamine | 5 | 2 | 1 | 93.8 | 28.16 |
| Phytol | 13 | 1 | 1 | 98.94 | 20.23 |
| Octadecanoic acid | 16 | 2 | 1 | 90.41 | 37.3 |
| 9,12,15-Octadecatrienoic acid, ethyl ester, (Z,Z,Z)- | 15 | 2 | 0 | 98.12 | 26.3 |
| 9-Decen-2-one | 7 | 1 | 0 | 49.91 | 17.07 |
| 1-Hentetracontanol | 39 | 1 | 1 | 200.36 | 20.23 |
| 7-Chloro-1-[[3-(dimethylamino)propyl]imino]-1,3,4,10-tetrahydro-10-hydroxy-3-(2-methoxyphenyl)-9(2H)-acridinone | 6 | 7 | 1 | 133.1 | 57.83 |
| 3-Trifluoroacetoxypentadecane | 15 | 5 | 0 | 85.31 | 26.3 |
| Squalene | 15 | 0 | 0 | 143.48 | 0 |
| Stigmasta-3,5-dien-7-one | 6 | 1 | 0 | 131.79 | 17.07 |

**Table. S4 Lipophilicity properties**

**Table.S5 Drug likeness characteristics of *R. repens* extract**

**Table. S6 Medicinal chemistry traits**

| ***R. repens compounds*** | **PAINS** | **Brenk** | **Leadlikeness** | **Synthetic accessibility** |
| --- | --- | --- | --- | --- |
| Phenol, 2,4-bis(1,1-dimethylethyl) | 0 | 0 | 2 | 1.34 |
| 2-Hexadecene,3,7,11,14-tetramethyl-,[R-[R*R*-(E)]]- | 0 | 1 | 2 | 4.24 |
| 2,6,6-trimethylbicyclo[3.1.1]heptane, (1.alpha.,2.beta.,5.alpha.)- | 0 | 0 | 2 | 4.03 |
| 1-Hexadecyne | 0 | 1 | 3 | 4.14 |
| Cyclohexanol, 1-ethynyl | 0 | 1 | 1 | 2.78 |
| 2-Pentadecanone, 6,10,14-trimethyl- | 0 | 0 | 2 | 3.2 |
| Bicyclo[2.2.1]heptane, 2,2,3-trimethyl | 0 | 0 | 2 | 3.2 |
| Acetic acid, 10,11-dihydroxy-3,7,11,-trimethyldodeca-2,6-denyl ester | 0 | 0 | 2 | 3.63 |
| Phthalic acid, butyl undecyl ester | 0 | 1 | 3 | 3.29 |
| N-[2-(Dimethylamino)ethyl]-2-phenyl-4-quinolinamine | 0 | 0 | 1 | 2.45 |
| Phytol | 0 | 1 | 2 | 4.3 |
| Octadecanoic acid | 0 | 0 | 2 | 2.54 |
| 9,12,15-Octadecatrienoic acid, ethyl ester, (Z,Z,Z)- | 0 | 0 | 2 | 2.54 |
| 9-Decen-2-one | 0 | 1 | 1 | 1.82 |
| 1-Hentetracontanol | 0 | 0 | 3 | 5.4 |
| 7-Chloro-1-[[3-(dimethylamino)propyl]imino]-1,3,4,10-tetrahydro-10-hydroxy-3-(2-methoxyphenyl)-9(2H)-acridinone | 0 | 2 | 2 | 4.84 |
| 3-Trifluoroacetoxypentadecane | 0 | 0 | 2 | 3.03 |
| Squalene | 0 | 0 | 2 | 3.03 |
| Stigmasta-3,5-dien-7-one | 0 | 0 | 2 | 6.37 |
